# Granulocyte colony stimulating factor use and adherence to clinical practice guidelines among women with breast cancer living in Puerto Rico: a population-based study

**DOI:** 10.1186/s12913-022-08325-3

**Published:** 2022-07-20

**Authors:** Yarixabeth Jiménez Nieves, Karen J. Ortiz-Ortiz, Ruth E. Ríos Motta, Maira A. Castañeda-Avila, Guillermo Tortolero-Luna

**Affiliations:** 1 Department of Health Services Administration, Graduate School of Public Health, Medical Sciences Campus, University of Puerto Rico, San Juan, Puerto Rico; 2 Division of Cancer Control and Population Sciences, University of Puerto Rico Comprehensive Cancer Center, PO Box 363027, San Juan, 00936-3027 Puerto Rico; 3 Puerto Rico Central Cancer Registry, University of Puerto Rico Comprehensive Cancer Center, San Juan, Puerto Rico; 4 Department of Population and Quantitative Health Sciences, University of Massachusetts Chan Medical School, Worcester, MA USA

**Keywords:** Febrile neutropenia, Granulocyte colony stimulating factor, Breast cancer, Health insurance, Clinical practice guidelines

## Abstract

**Background:**

Febrile Neutropenia (FN) is a common and serious condition related to cancer chemotherapy. Human recombinant Granulocyte-Colony Stimulating Factor (G-CSF) prevents and attenuates the severity and duration of FN. We evaluated the use and predictors of G-CSF adherence among women with breast cancer with a high risk of FN in Puerto Rico.

**Methods:**

This retrospective cohort study used the Puerto Rico Central Cancer Registry-Health Insurance Linkage Database. Women with invasive breast cancer diagnosed during 2009-2015 who received selected chemotherapy regimens (*n* = 816) were included. The risk of FN was categorized as high and low risk based on the chemotherapy regimens according to the National Comprehensive Cancer Network guidelines and literature. *Adherence* was defined as the use or no use of G-CSF at the start of the first chemotherapy cycle among women with breast cancer based on the risk of developing FN. We used a multivariate logistic model to identify factors associated with G-CSF use in women classified at high risk for FN.

**Results:**

Adherence to G-CSF clinical practice guidelines was low (38.2%) among women with a high risk of FN. Women at high risk of FN with Medicaid (aOR: 0.14; CI 95%: 0.08, 0.24) and Medicare/Medicaid (aOR: 0.33; CI 95%: 0.15, 0.73) were less likely to receive G-CSF than women with private health insurance. Women with regional stage (aOR: 1.82; CI 95%: 1.15, 2.88) were more likely to receive G-CSF than women with localized cancers.

**Conclusions:**

Adherence to clinical practice guidelines was poor among women with a high risk of FN. Furthermore, disparities in the adherence to G-CSF use in terms of health insurance, health region, and cancer stage granted the opportunity to implement strategies to follow the recommended guidelines for using G-CSF as part of cancer treatment.

## Introduction

Breast cancer is treatable and even curable if detected promptly and after receiving treatments, like chemotherapy and radiation [1]. However, it is common for various chemotherapy regimens to induce serious conditions like febrile neutropenia (FN) [2–5]. When experiencing FN, a simple episode of fever may require hospitalization and antibiotics. Studies have shown that FN is the major dose-limiting toxicity of chemotherapy regimens [6]. Treatment for FN often requires a reduction in chemotherapy dose sessions or treatment delay [7, 8]. Reduction in chemotherapy dose intensity is associated with disease recurrence and mortality [6]. Patients with FN often cannot continue with their treatment until their immune system recovers, and dosing reductions or delays in chemotherapy can affect cancer treatment success, mainly when treatment intent is curative [6].

Granulocyte-Colony Stimulating Factors (G-CSFs) are biological growth factors that support proliferation, differentiation, and activation of granulocytes [9] and can attenuate the severity and duration of FN associated with systemic chemotherapy [6]. The National Comprehensive Cancer Network (NCCN) clinical practice guidelines recommend the use of G-CSF when the anticipated risk of FN associated with chemotherapy is 20% or higher (high-risk). Likewise, G-CSF use is recommended when the risk is 10 to 20% (intermediate-risk) and the patient has additional risk factors. Meanwhile, G-CSF is not recommended when the risk is less than 10% (low-risk) [10]. Despite guidelines for G-CSF administration, the use of these agents in a clinical setting is inconsistent [11]. Differences in the use (overuse, underuse, or misuse) of G-CSF are related to physician and patient factors [11]. Studies have shown that G-CSF is underutilized in patients undergoing chemotherapy treatments associated with a high risk of developing FN while over-utilized in patients with a low risk of developing FN [11, 12].

To our knowledge, there are no studies evaluating the patterns of G-CSF use among Hispanics in the United States of America. The lack of information about the use of the G-CSF makes this study imperative since it provides valuable information about how G-CSF is being used among Hispanic women with breast cancer in Puerto Rico. Therefore, this study evaluates the patterns of use of G-CSF at the start of the first chemotherapy cycle and the adherence to clinical practice guidelines in women with a breast cancer diagnosis reported for the period 2009-2015 in Puerto Rico.

## Methods

### Data source

Data were obtained from the Puerto Rico Central Cancer Registry-Health Insurance Linkage Database (PRCCR–HILD). The PRCCR-HILD contains clinical and demographic data for cancer cases from the Puerto Rico Central Cancer Registry (PRCCR). The PRCCR is a *Gold Certified Registry* by the North American Association of Cancer Registries (NAACR) and recognized as *a Registry of Distinction* by the National Program of Cancer Registries (NPCR) [13]. The PRCCR database was linked with health insurance claim data provided by health insurance companies. PRCCR-HILD includes information for approximately 90% of Puerto Rico’s cancer cases from 2008 to 2017, allowing us to examine the utilization of health services among cancer patients in Puerto Rico. As a part of the development of the PRCCR-HILD, a deterministic match using a similar algorithm to the one used by SEER-Medicare was performed. All data were de-identified to ensure that no protected health information could be linked to individual patients. This study was reviewed and approved by the Institutional Review Board of the University of Puerto Rico, Medical Sciences Campus, San Juan, Puerto Rico.

### Study population

The study population consisted of women 21 years of age or older, residents of Puerto Rico with a diagnosis of invasive breast cancer (excluding lymphomas and sarcomas.) during the period 2009-2015, included in the PRCCR-HILD and who received selected chemotherapy regimens as part of the treatment plan (Table 1). We excluded patients with previously invasive cancer, patients with invalid diagnosis dates, and patients with unknown age at diagnosis.

**Table 1:** Febrile neutropenia risk according of chemotherapy regimens and guidelines recommendations for used of granulocyte colony stimulating factor

Febrile neutropenia risk	Agent or Combination of Agent	NCCN guidelines for the use of G-CSF
High (> 20%)	• TAC (Docetaxel, Doxorubicin and Cyclophosphamide) every 21 days.	Recommend prophylactic use of G-CSF
	• TC (Docetaxel and Cyclophosphamide) every 21 days.	
	• Dose dense AC followed by Taxanes: (Doxorubicin or Adriamycin plus Cyclophosphamide) followed by Paclitaxel or Docetaxel) every 14 days.	
	• TEC (Paclitaxel /Docetaxel, Epirubicin and Cytoxan) every 21 days.	
	• CMF (IV Cyclophosphamide, Methotrexate, and Fluorouracil) every 21 days.	
	• Paclitaxel every 21 days	
	• FEC (Fluorouracil, Epirubicin and Cyclophosphamide) plus sequential Docetaxel every 21 days	
	• Doxorubicin every 21 days	
	• Docetaxel every 21 days	
Low (< 10%)	• FAC (Fluorouracil, Doxorubicin and Cyclophosphamide) every 21 days	Routine use of G-CSF is not recommended.
	• Gemcitabine8 every 28 days	
	• Paclitaxel4 weekly	
	• EC: Epirubicin and Cyclophosphamide) every 21 days	
	• Docetaxel weekly	
	• Doxorubicin weekly	
	• Cyclophosphamide every 28 days	

### Study variables

#### Risk to develop febrile neutropenia

Patients were stratified as having a high (> 20%) or low (< 10%) risk of developing FN. This stratification was according to the chemotherapy regimen received based on NCCN guidelines, board-certified oncologist’s consensus, and peer-reviewed publications (Table 1) [10]. The intermediate-risk group was not considered for the evaluation of adherence to clinical practice guidelines since the patient’s risk factors, such as prior episodes of FN, poor nutritional status, the presence of open wounds or active infections, cytopenia due to bone marrow involvement by tumor, among others, were not available through claims data. Healthcare Common Procedure Coding System (HCPCS) was used to determine the active agent of the chemotherapy regimen. The algorithm considered the chemotherapeutic agents and the lapse in which they were administered.

#### Utilization and adherence to guidelines for the use of G-CSF

The use of G-CSF as primary prophylaxis was evaluated at the start of the chemotherapy regimen. Following a previous study [2], we considered G-CSF (filgrastim, pegfilgrastim, or sargramostim) as primary prophylaxis if it was used within 7 days after the first chemotherapy cycle.

*Adherence* to clinical practice guidelines for G-CSF use was defined as follows: 1) adherent: patients with a high risk of FN who received G-CSF 7 days after the first chemotherapy administration; 2) non-adherent: patients with a high risk of FN who did not receive G-CSF 7 days after the first chemotherapy administration [2]. Guidelines for these patients consider the provider’s appraisal of additional patient characteristics for G-CSF administration [10]. Since a low number of women with breast cancer were classified as having a low risk of FN, we were unable to evaluate factors associated with adherence to clinical practice guidelines.

#### Independent variables

This study used the adaptation of Anderson and Newman’s Framework of Health Services Utilization to identify the predictors of adherence to guidelines for the use of G-CSF [14]. The framework’s core idea is to identify the conditions that either facilitate or impede the use of health services [14] which are the determinants of the use of G-CSF as part of the patient’s breast cancer treatment. The independent variables were age group (21-49, 50-64, ≥65), marital status (married, not married), cancer stage (localized, regional, distant, unknown), Puerto Rico Department of Health Region (Metro, Arecibo, Bayamón, Caguas, Fajardo, Mayagüez, Ponce), type of health insurance (Medicaid, Medicare, Medicaid/Medicare and private), and comorbidities. Additionally, we used the National Cancer Institute Comorbidity Index to assess comorbidities [15].

### Statistical analysis

Descriptive statistics were used to describe the sociodemographic characteristics of women with breast cancer receiving chemotherapy. Logistic regression models were used to estimate crude odds ratios (ORs), adjusted odds ratios (aORs), and 95% confidence intervals (CIs). Patients with missing data were excluded from logistic regression models. We examined predisposing, enabling, and need factors associated with G-CSF clinical guidelines adherence. Variables selected for multivariable analysis were limited to those significantly related to G-CFS use and the other predictor variables to avoid over-adjustment in the adjusted model. Model fits were examined using the likelihood ratio test, Bayesian information classification (BIC), and Akaike information classification (AIC). The likelihood ratio test statistic was used to assess the significance of interaction terms. Statistical analyses were performed using Stata/SE version 16.0 statistical software (Stata Corp., LP., College Station, TX).

## Results

A total of 816 women with breast cancer were included in this analysis. More than two-thirds of women were 50 years or older (67.8%), half were married (51.6%), nearly half had private insurance (44.2%), and nearly half had a regional stage at diagnosis (44.5%) (Table 2).

**Table 2:** Socio-demographic characteristics of the study population (816)

Characteristics	n	%
**Predisposing factors**		
***Age at diagnosis***		
21-49	263	23.2
50-64	334	40.9
≥ 65	219	26.8
***Marital status***		
Married	386	47.3
Unmarried	421	51.6
Unknown	9	1.1
**Enabling factors**		
***Health insurance***		
Private insurance	361	44.2
Medicaid	243	29.8
Medicare	92	11.3
Duals (Medicare & Medicaid)	120	14.7
***Health region***		
Mayagüez	118	14.5
Arecibo	94	11.5
Bayamón	121	14.8
Ponce	140	17.2
Metro	199	24.4
Fajardo	31	3.8
Caguas	113	13.9
**Need factors**		
***Cancer stage***		
Local	366	44.9
Regional	363	44.5
Distant	73	9.0
Unknown	14	1.7
***NCI Comorbidity Index***		
0	673	82.5
1	73	9.0
≥ 2	70	8.6

### Use of granulocyte colony stimulating factors

**Fig. 1:**
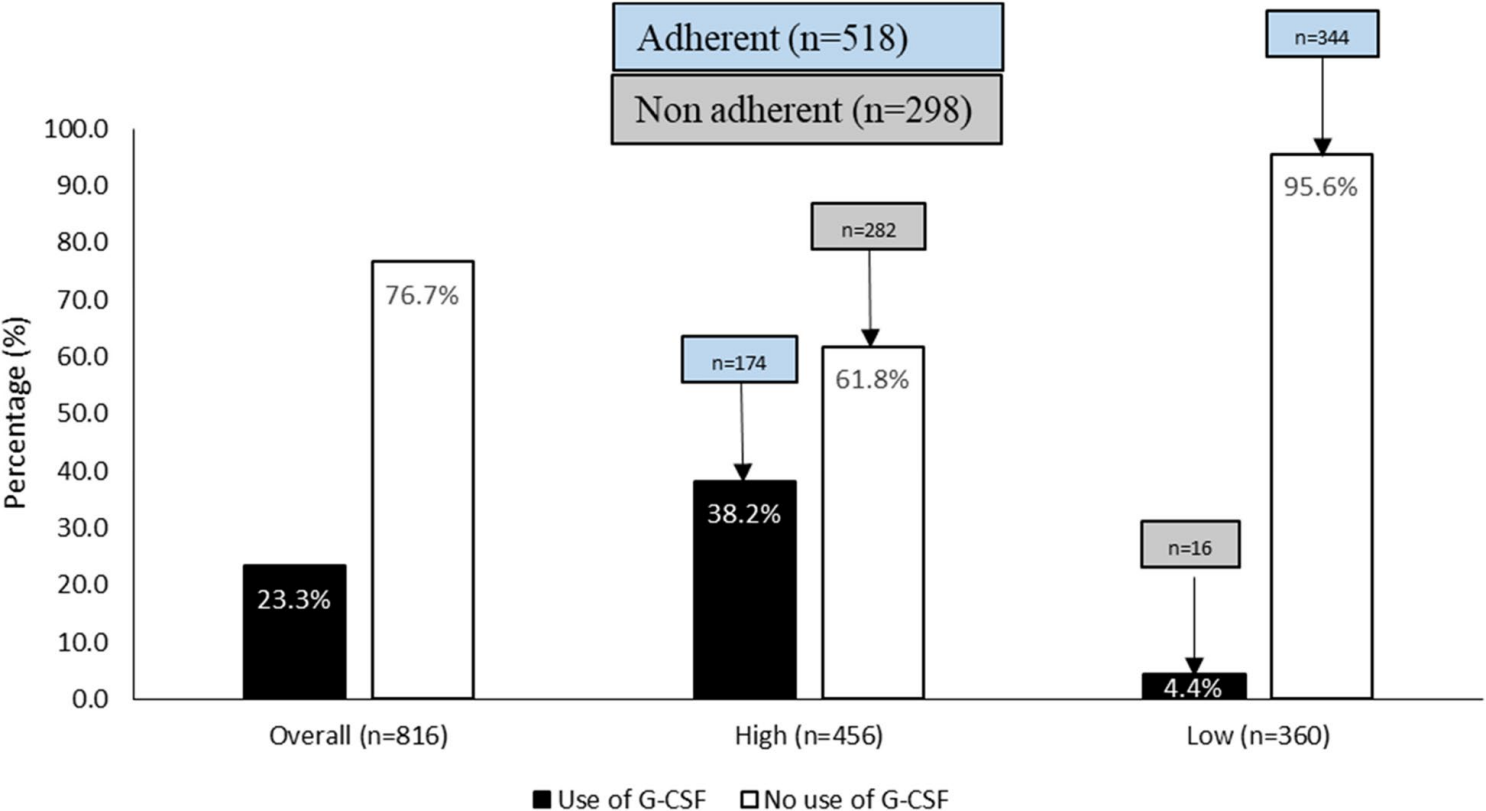
Use of granulocyte colony stimulating factor by febrile neutropenia risk level among the study population (*n* = 816)

Of those women with a low risk of FN, 4.4% received G-CSF. Among women with high risk of FN, 38.2% received G-CSF (Fig. 1). We performed an analysis of adherence to clinical guidelines in using G-CSF. A total of 298 women (36.5%) were non-adherent to G-CSF guidelines (282 women had a high-risk and 16 women had a low risk of developing FN), and 63.5% (*n* = 518) followed the recommendation on how to use G-CSF based on the risk of developing FN (Fig. 1).

### Factors associated with adherence to G-CSF clinical practice guidelines

After adjusting for all other variables, we found that women 65 years or older at the time of breast cancer diagnosis were less likely to be adherent to G-CSF guidelines than women 21-34 years old. Additionally, women with Medicaid were 86% less likely to be adherent to G-CSF guidelines than women with private health insurance (aOR: 0.14, 95% CI: 0.08-0.24). Compared with all health regions, women living in the Metro Region were more likely to be adherent to G-CSF guidelines than those in other regions. In addition, women with regional stage cancer were 82% (aOR: 1.82, 95% CI: 1.15-2.88) more likely to be adherent to G-CSF guidelines than women with localized cancer stage (Table 3).

**Table 3:** Factors associated with adherence to G-CFS clinical practice guidelines among the study population receiving high-risk chemotherapy

Factors	Unadjusted Odds Ratios (IC 95%)	Adjusted Odds Ratios (IC 95%)^**c**^
**Predisposing factors**		
***Age***		
21–49	Reference	Reference
50–64	0.61 (0.40–0.94)	0.64 (0.38–1.06)
≥ 65	0.41 (0.24–0.69)	0.43 (0.20–0.91)
***Marital Status***^***a***^		Excluded
Married	1.27 (0.87–1.87)	
Unmarried	Reference	
**Enabling factors**		
***Health insurance***		
Private	Reference	Reference
Medicaid	0.15 (0.09–0.25)	0.14 (0.08–0.24)
Medicare	0.38 (0.20–0.73)	0.59 (0.26–1.35)
Duals (Medicare &Medicaid)	0.24 (0.12–0.45)	0.33 (0.15–0.73)
***Health region***		
Metro	Reference	Reference
Arecibo	0.16 (0.06–0.43)	0.11 (0.04–0.34)
Bayamón	0.37 (0.20–0.66)	0.35 (0.18–0.68)
Caguas	0.41 (0.21–0.79)	0.62 (0.30–1.28)
Fajardo	0.34 (0.13–0.89)	0.50 (0.17–1.46)
Mayagüez	0.28 (0.15–0.55)	0.29 (0.14–0.61)
Ponce	0.44 (0.25–0.79)	0.51 (0.27–0.98)
**Need factors**		
***Cancer stage***^***b***^		
Localized	Reference	Reference
Regional	1.51 (1.01–2.25)	1.82 (1.15–2.88)
Distante	1.73 (0.84–3.56)	2.12 (0.92–4.90)
***NCI Comorbidity Index***		Excluded
0	Reference	
1	0.45 (0.21–0.98)	
≥ 2	0.53 (0.24–1.16)	

^a^Cases with marital status unknown were excluded

^b^Cases with stage unknown were excluded

^c^Marital status and NCI comorbidity index were excluded from the adjusted model

## Discussion

This study evaluated G-CSF use administered at the start of the first chemotherapy cycle and the adherence to clinical practice guidelines in women with a breast cancer diagnosis in Puerto Rico. We found that a high percentage of women (61.8%) with a high risk for FN did not receive the recommended G-CSF as part of their treatment. The underuse of G-CFS among breast cancer patients in Puerto Rico with a high risk for FN is concerning because it may affect patient outcomes. Moreover, the results provided by this study evidenced variations in the use of G-CSF, suggesting inequities that should be addressed.

Findings from previous studies were congruent to our findings, where it has been found that G-CSF is underutilized mainly due to the high costs of these treatments, and cost-effectiveness heavily relies on the individual risk of FN [11, 16]. The factors for the G-CFS underuse in Puerto Rico remain to be determined, but factors related to the physician, the healthcare setting, or the patient have been identified in other studies [11, 17–19]. However, other contextual factors in Puerto Rico could influence G-CSF underutilization, where only 38.2% of the patients at high risk of developing FN followed clinical practice guidelines. Puerto Rico, a territory of the United States of America, has 3.3 million people, where women represent 53% of the population. Nearly 44% of Puerto Rico’s women are 50 years or older, and 45% live below the poverty level [20]. In addition, almost half of Puerto Rico’s population (46%) has Medicaid, a federal and state-funded insurance program that provides insurance coverage to low-income people of every age. The socioeconomic conditions in Puerto Rico, including the fiscal crisis with a default on its debt, and professional health shortages, could negatively impact access and use of health services [21, 22]. Furthermore, a sequence of multiple disasters in previous years, including extreme hurricanes, earthquakes, and the COVID-19 pandemic, could have worsened the access to high-quality care. Therefore, future studies are warranted to better elucidate specific barriers to using G-CFS appropriately.

In addition to the underuse of G-CFS among breast cancer patients with a high risk for FN, we found that enabling factors, including health insurance and health region, were strongly associated with G-CSF use. Patients with private health insurance were most likely to be adherent to guidelines for the use of G-CSF, followed by Medicare patients. Medicaid patients and dually eligible patients (Medicaid/Medicare) were the categories less likely to be adherent to guidelines for G-CSF use. Similar to our findings, previous studies have shown that patients with high-risk chemotherapy regimens with private health insurance plans are more likely to receive G-CSF, contrary to patients with Medicare or Medicaid insurance coverage [23]. These findings are relevant, given that almost half of Puerto Rico’s population (46%) has Medicaid. This result highlights the presence of health disparities by health insurance type. Therefore, further studies evaluating factors that explain these disparities, including variations in the quality of services and healthcare coverage are required.

Moreover, the health region was strongly associated as a predicting factor for G-CSF guidelines adherence. Patients in the Metro region were most likely to adhere to G-CSF guidelines. This finding may be attributed to larger cancer centers and a higher concentration of specialized health professionals for cancer treatment in this region [21]. In fact, in Puerto Rico, only three institutions are accredited by the Commission on Cancer (CoC). The CoC accreditation encourages hospitals, treatment centers, and other facilities to improve care through cancer-related programs and activities [24].

Other factors associated with adherence to guidelines for the use of G-CSF include stage of diagnosis. Women diagnosed at regional stage were more likely to adhere to guidelines for G-CSF use. Literature has shown that breast cancer patients in the regional stage with larger tumor size and greater node positivity are more likely to receive G-CSF [11]. In terms of predisposing factors, clinical guidelines recommend G-CSF use on patients under high-risk chemotherapy regimens, and older-aged are considered risk factors for severe FN complications [25]. However, we found that women 65 years or older were less likely to be adherent to G-CSF guidelines than younger women.

Consequently, variations in the adherence to G-CSF use among women with breast cancer are not explained by health needs but by social and economic factors. These results add to previous literature suggesting that differences in disadvantaged groups, such as the poor, racial minorities, women, or other groups who have persistently experienced social disadvantage or discrimination, systematically experience worse health or greater health risks than more advantaged social groups [26, 27]. Access to care is vital for promoting the health of the population and achieving equity. Social disparities and inequalities in the provision of health care can impact both the patient’s health outcomes and costs related to potentially avoidable care. These types of disparities leave some patients with a higher burden of disease and mortality [28].

The results provided by this study suggest inequalities that would require interventions that facilitate access to the appropriate use of G-CSF. In fact, the SWOG Cancer Research Network (SWOG) conducted a pragmatic cluster-randomized cancer care delivery trial where Puerto Rico participated. This trial evaluated the effectiveness of an order prescribing intervention to improve CSF use for cancer patients receiving chemotherapy [29]. This intervention trial has the goal to help physicians decide whether to prescribe CSF and provide changes in the prescription order system [29]. The results of this study may provide new evidence to improve the appropriate use of G-CFS. Nevertheless, our findings provide guidance for future interventions that should consider the disparities in adherence to G-CSF among patients with Medicaid, older patients, regional stage at diagnosis, and living in non-metropolitan regions of Puerto Rico.

### Strengths and limitations

The absence of studies that address this topic makes this work a relevant contribution to the scientific literature since no studies have been published that address adherence to G-CFS guidelines among women with breast cancer in Puerto Rico. In addition, the PRCCR-HILD database allows us to generalize the findings to the population of women with breast cancer in Puerto Rico. Given that the selection process and sample size are representative of the general population, the database collects all reported cases on the island. Whereas, some limitations of the study include different operational definitions of G-CSF administration time encountered across the scientific literature; this makes it difficult to compare the study results with other studies. Also, we could not assess previous diagnoses of FN, bone marrow involvement by tumor, recent surgery or open wounds, and liver or renal dysfunction. Those are risk factors for G-CSF use and are tied to clinical practice guidelines as an evaluation requirement for prescribing the G-GFS among patients with intermediate-risk of febrile neutropenia. The lack of this information limited our study to evaluate adherence only among those with a low and high risk of FN. We also could not assess additional important socioeconomic variables, including employment, education, and income. Lastly, G-CSF and chemotherapy prescription timing were determined by medical claims, and errors due to coding inaccuracies may have been introduced.

## Conclusion

Our findings indicate that adherence to clinical practice guidelines for G-CFS use is unequal across the population. We found a high percentage of women with breast cancer in Puerto Rico with a low risk of FN adhering to the G-CFS use guidelines. Meanwhile, adherence to clinical practice guidelines was poor among the women with a high risk of FN. This finding is crucial since it underscores that the group considered the most compromised in health conditions and risk was the group left with an unattended need. Moreover, differences in adherence were observed in terms of health insurance, age, health region, and cancer stage. Patients with private insurance, younger patients, those from the Metro region, and those with regional cancer stage were more adherent to the guidelines for using G-CSF compared to their counterparts. The findings of this study showed that within the healthcare system, there is a possible pattern of disparity that granted the opportunity to implement strategies to follow the recommended guidelines for using G-CSF as part of cancer treatment.

## Data Availability

The datasets generated and analyzed during the current study are not publicly available due to the confidentiality policy of the Puerto Rico Central Cancer Registry but are available from the corresponding author on reasonable request.

## Abbreviations

AOR: Adjusted Odds Ratio
CI: Confidence Interval
CPT: Current Procedural Terminology
FN: Febrile Neutropenia
G-CSF: Granulocyte Colony Stimulating Factor
HCPCS: Healthcare Common Procedure Coding System
ICDO-3: International Classification of Diseases for Oncology 3rd Edition
NCCN: National Comprehensive Cancer Network
NDC: National Drug Code
NOS: No Other Specified
OR: Odds Ratio
PR: Puerto Rico
PRCCR: Puerto Rico Central Cancer Registry
PRCCR–HILD: Puerto Rico Central Cancer Registry-Health Insurance Linkage Database
SEER-Medicare: Surveillance, Epidemiology and End Results-Medicare

## References

[1] McDonald ES, Clark AS, Tchou J, Zhang P, Freedman GM (2016). Clinical diagnosis and management of breast cancer. J Nucl Med.

[2] Ramsey SD, Mccune JS, Blough DK, Mcdermott CL, Clarke L, Malin JL (2010). Colony-stimulating factor prescribing patterns in patients receiving chemotherapy for cancer.

[3] Chia VM, Page JH, Rodriguez R, Yang SJ, Huynh J, Chao C (2013). Chronic comorbid conditions associated with risk of febrile neutropenia in breast cancer patients treated with chemotherapy. Breast Cancer Res Treat.

[4] Lucas AJ, Olin JL, Coleman MD (2018). Management and preventive measures for febrile neutropenia. P T.

[5] Teng TS, Ji AL, Ji XY, Li YZ (2017). Neutrophils and immunity: from bactericidal action to being conquered. J Immunol Res.

[6] Cooper KL, Madan J, Whyte S, Stevenson MD, Akehurst RL (2011). Granulocyte colony-stimulating factors for febrile neutropenia prophylaxis following chemotherapy: systematic review and meta-analysis.

[7] Căinap C, Cetean-Gheorghe S, Pop LA, Leucuta DC, Piciu D, Mester A (2021). Continuous intravenous administration of granulocyte-colony-stimulating factors—a breakthrough in the treatment of cancer patients with febrile neutropenia. Medicina.

[8] Ward AS, Kabiri M, Yucel A, Silverstein AR, van Eijndhoven E, Bowers C (2019). The long-term social value of granulocyte colony-stimulating factors. Am J Manag Care.

[9] Horiuchi T, Shimizu K, Sasaki K, Kato A, Homma Y (2017). Granulocyte-colony stimulating factor producing infiltrating urothelial carcinoma of the left renal pelvis: a case report. Urol Case Rep.

[10] Griffiths EA, Chair V, Alwan L, Bachiashvili K, Benrashid M, Brown A (2019). Continue NCCN guidelines panel disclosures NCCN guidelines version 2.2019 hematopoietic growth factors.

[11] Barnes G, Pathak A, Schwartzberg L (2014). G-CSF utilization rate and prescribing patterns in United States: associations between physician and patient factors and GCSF use. Cancer Med.

[12] Okunaka M, Kano D, Matsui R, Kawasaki T, Uesawa Y (2021). Comprehensive analysis of chemotherapeutic agents that induce infectious neutropenia. Pharmaceuticals.

[13] Torres-Cintrón C, Alvarado-Ortiz M, Román-Ruiz Y, Ortiz-Ortiz K, Zavala-Zegarra D, Tortolero-Luna G. Cancer in Puerto Rico Report, 2014-2018. San Juan, PR; 2021.

[14] Aday L, Begley C, Lairson D, Slater C (2004). Evaluating the healthcare system: effectiveness, efficiency, and equity.

[15] National Cancer Institute (2021). NCI comorbidity index overview.

[16] Neutropenia F (2017). The value of granulocyte colony-stimulating factors in managing febrile neutropenia.

[17] Bennett CL, Bishop MR, Tallman MS, Somerfield MR, Feinglass J, Smith TJ (1999). The association between physician reimbursement in the US and use of hematopoietic colony stimulating factors as adjunct therapy for older patients with acute myeloid leukemia: results from the 1997 American Society of Clinical Oncology survey. Health Services Research Committee of the American Society of Clinical Oncology. Ann Oncol Off J Eur Soc Med Oncol.

[18] Bennett CL, Weeks JA, Somerfield MR, Feinglass J, Smith TJ (1999). Use of hematopoietic colony-stimulating factors: comparison of the 1994 and 1997 American Society of Clinical Oncology surveys regarding ASCO clinical practice guidelines. Health Services Research Committee of the American Society of Clinical Oncology. J Clin Oncol.

[19] Potosky AL, Malin JL, Kim B, Chrischilles EA, Makgoeng SB, Howlader N (2011). Use of colony-stimulating factors with chemotherapy: opportunities for cost savings and improved outcomes. J Natl Cancer Inst.

[20] US Census Bureau (2019). American community survey 1-year estimates.

[21] Perreira K, Lallemand N, Napoles A, Zuckerman S (2017). Environmental scan of Puerto Rico’s health care infrastructure.

[22] Roman J (2015). The Puerto Rico healthcare crisis. Ann Am Thorac Soc.

[23] Sullivan SD, Ramsey SD, Blough DK, McDermott CL, Clarke L, McCune JS (2011). Health care use and primary prophylaxis with colony-stimulating factors. Value Health.

[24] Voelker. Cancer Care Accreditation Standards: Improve Quality and Help Patients Cope. JAMA. 2011;306(12):1314. 10.1001/jama.2011.1373 21954472

[25] Lyman GH, Kuderer N, Agboola O, Balducci L (2003). Evidence-Based Use of Colony-Stimulating Factors in Elderly Cancer Patients. Cancer Control..

[26] Braveman P (2006). Health disparities and health equity: concepts and measurement. Annu Rev Public Health.

[27] Thomas B (2014). Health and health care disparities: the effect of social and environmental factors on individual and population health. Int J Environ Res Public Health.

[28] Tortolero-Luna G, Torres-Cintrón CR, Alvarado-Ortiz M, Ortiz-Ortiz KJ, Zavala-Zegarra DE, Mora-Piñero E (2019). Incidence of thyroid cancer in Puerto Rico and the US by racial/ethnic group, 2011-2015. BMC Cancer.

[29] Bansal A, Sullivan SD, Hershman DL, Lyman GH, Barlow WE, Mccune JS (2017). A stakeholder-informed randomized, controlled comparative effectiveness study of an order prescribing intervention to improve colony stimulating factor use for cancer patients receiving myelosuppressive chemotherapy: the TrACER study. J Comp Eff Res.

